# Cooperative Interactions between Different Classes of Disordered Proteins Play a Functional Role in the Nuclear Pore Complex of Baker’s Yeast

**DOI:** 10.1371/journal.pone.0169455

**Published:** 2017-01-09

**Authors:** David Ando, Ajay Gopinathan

**Affiliations:** 1 Physical Biosciences Division, Lawrence Berkeley National Laboratory, Berkeley, CA, United States of America; 2 Joint BioEnergy Institute, Emeryville, CA, United States of America; 3 Department of Physics, University of California, Merced, CA, United States of America; University of Minnesota Twin Cities, UNITED STATES

## Abstract

Nucleocytoplasmic transport is highly selective, efficient, and is regulated by a poorly understood mechanism involving hundreds of disordered FG nucleoporin proteins (FG nups) lining the inside wall of the nuclear pore complex (NPC). Previous research has concluded that FG nups in Baker’s yeast (*S. cerevisiae*) are present in a bimodal distribution, with the “Forest Model” classifying FG nups as either di-block polymer like “trees” or single-block polymer like “shrubs”. Using a combination of coarse-grained modeling and polymer brush modeling, the function of the di-block FG nups has previously been hypothesized in the *Di-block Copolymer Brush Gate* (DCBG) model to form a higher-order polymer brush architecture which can open and close to regulate transport across the NPC. In this manuscript we work to extend the original DCBG model by first performing coarse grained simulations of the single-block FG nups which confirm that they have a single block polymer structure rather than the di-block structure of tree nups. Our molecular simulations also demonstrate that these single-block FG nups are likely cohesive, compact, collapsed coil polymers, implying that these FG nups are generally localized to their grafting location within the NPC. We find that adding a layer of single-block FG nups to the DCBG model increases the range of cargo sizes which are able to translocate the pore through a cooperative effect involving single-block and di-block FG nups. This effect can explain the puzzling connection between single-block FG nup deletion mutants in *S. cerevisiae* and the resulting failure of certain large cargo transport through the NPC. Facilitation of large cargo transport via single-block and di-block FG nup cooperativity in the nuclear pore could provide a model mechanism for designing future biomimetic pores of greater applicability.

## Introduction

**Fig 1:**
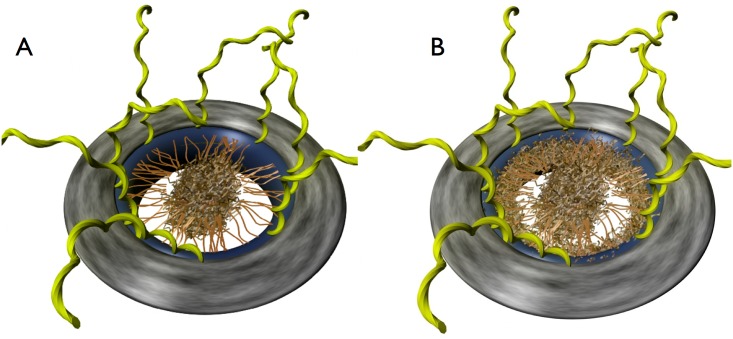
Illustration of DCBG models. Each of the Nuclear Pore Complexes depicted has a cylindrical structural domain (navy blue) which spans the lipid bilayers (grey) which make up the nuclear envelope, oriented such that the tops of the pores face the cytoplasm, while the bottoms of the pores face the nucleus. The nuclear basket, located on the nuclear face of the pore is not shown for simplicity. Additionally the cytoplasmic filaments of the pore, which are not directly responsible for gating [10], are shown in yellow. A: In the original DCBG model individual di-block FG nups (brown) have collapsed coil gel-like regions (dark brown) and extended coil brush-like domains (light brown), resulting in a microphase separation of these domains within the NPC. This results in a central plug-like structure supported by a polymer brush of extended disordered regions of FG nups. [11] B: The modified DCBG model which is the focus of this manuscript is the same as in part A except for the addition of a dense region of single-block FG nups which lie along the wall of the NPC. The core of our proposed Di-block Copolymer Brush Gate model is the idea that when particular transport factors are present which are able to outcompete the inter-FG domain “sticky tip” interactions, the polymer brush which fills the NPC is able to open up to a new free energy minimum that can accommodate the transit of cargo, while when interactions between sticky tips are able to recover into the several kT range, the pore is able to close.

In eukaryotes the nuclear pore complex (NPC) is responsible for regulating all traffic which moves across the nuclear envelope. The NPC spans the nuclear envelope from the cytoplasmic face to the nuclear side of the envelope, extending, ∼35–50 nm across, and controls important processes such as the import of regulatory proteins from the cytoplasm and the export of RNA from the nucleus [1–5]. Roughly thirty different types of NPC proteins (nups) make up the NPC protein complex, and these different protein types are present in multiples of eight given the eightfold symmetry of the pore itself [2]. Some nups form a structural cylindrical like structure that is embedded within the nuclear envelope. This cylindrical structure forms a channel ∼50–80 nm in diameter that connects the nucleoplasm and cytoplasm. A second set of nups (orthogonal to the structural nups), and which are the main target of study in this paper, are disordered proteins which are grafted to the inside wall of the NPC channel and which are responsible for forming a selective diffusion barrier to transport [1]. Containing several phenylalanine-glycine (FG) amino acid repeats each, these nups are referred to as “FG nups” and are intrinsically disordered [6]. FG repeats themselves separate into different classes, with well studied GLFG and FxFG repeat motifs known to possess different binding affinities to the various nuclear transport factors [7]. In common Baker’s yeast, *Saccharomyces cerevisiae*, every individual nuclear pore has around 150 of the disordered type FG nups total which create a polymeric region within the pore that functions as a semi-permeable diffusion barrier, Fig 1. This barrier is able to actively regulate transport for particles larger than roughly 4 nm in size, whereas particles smaller than this size are allowed to passively diffuse through the pore in an unregulated manner. Amazingly, the NPC allows for active transport of cargoes up to ∼40 nm in size [8], over short timescales of 5–20 ms per transported cargo [9]. Additionally the NPC does not directly expend any energy to regulate transport, although it does use energy in the form of transport gating free energy consumption, which is limited to the maintenance of a gradient of transport factors across the nuclear membrane.

Although the NPC plays a key role in eukaryotic biology and numerous studies have been performed to determine the structure and properties of individual nucleoporins [1–3, 6, 12–15], the structure of the polymer complex composed of disordered FG nups within the NPC and its precise mechanism of transport regulation remain unclear. Different models for NPC structure and function, such as the “hydrogel” [16] and “virtual gate” [17] models, assume very different morphologies for the complex of disordered proteins which fills the nuclear pore. For example, the hydrogel model [18] predicts that FG nups interact via hydrophobic amino acids to form a dense filamentous meshwork that physically blocks protein diffusion while the virtual gate model predicts that FG nups have limited hydrophobic interactions and instead take on an extended polymer brush like structure that form an entropic gate at the NPC which blocks protein diffusion. Another model, the *Di-block Copolymer Brush Gate* (DCBG) model [11], Fig 1A, assumes that the key FG nups which regulate transport are individually bi-phasic, while most theoretical and polymer physics approaches [19–22] to the NPC transport problem tend to assume a homogenous structure for individual FG nups, resulting in a relatively homogenous NPC architecture. More recent advances in coarse grained simulations of the nucleoporins that consider sequence have alleviated this issue [23–29] but still lack the level of detail necessary to produce potential secondary or tertiary structure. More importantly, none of the theory or simulations to date have really shed light on the specific roles of the different types of FG nups within a single NPC and the functional reason for their co-existence. In fact, some models and experiments actually imply that the amino acid sequence and structure of the FG nups and transport factors may not be critical in regulating the transport of cargo across the NPC [28, 29].

**Fig 2:**
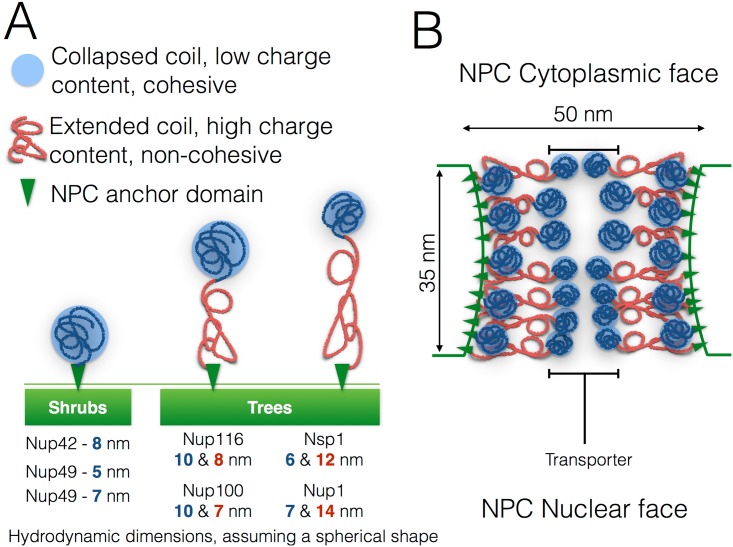
Biphasic FG nups and illustration of the Forest model. A: Diagrams of the various FG nups in *S. cerevisiae* and their hydrodynamic radii. Blue or red lines depict either high (red) or low (blue) content of charged AAs along the disordered region of the FG nup. The small green triangles represent the anchor domain of each FG nup. Single-block FG nups, termed “shrubs” in the Forest model, were categorized as consisting of a continuos collapsed FG domain adjacent to an anchor domain, consisting of Nup57, Nup49, and Nup42. Di-block FG nups, or “trees” in the terminology of the Forest model, were categorized as having a collapsed FG domain at their free ends separated from the anchor domain by a extended coil domain B: A diagram of the Forest model NPC architecture. Orientation of the pore is such that top side faces the cytoplasm, while the bottom side faces the nucleus. FG nups are drawn to scale and positioned according to the relative location of their anchor domains along the z-axis of the NPC, as determined by immuno-localization [30].

The original DCBG model is based off a previous analysis by Yamada *et al* [30] which showed the existence of di-block FG nups within the pore. Yamada *et al* also reported that the different types of FG nups from Baker’s yeast are present in a bi-modal distribution *in vivo*, with some FG nups forming di-block “trees” while others form single-block “shrubs”, Fig 2A. The di-block tree FG nups have one disordered region near the end of the nup which is grafted to the pore wall that resembles an extended polymer and contains a high density of charged amino acids, while di-block tree FG nups have another disordered region at their free end which is collapsed upon itself and contains numerous FG repeats and relatively few charged amino acids. In the DCBG model these bi-phasic “tree” FG nups form a copolymer brush within the pore which fills the NPC (Fig 1A), where the approach of certain forms of cargo can alter the local brush structure, effectively opening the closed brush structure and allowing for transport. In the original Forest model of NPC structure by Yamada *et al* [30] Fig 2B, single-block FG nups localize to the pore wall, in addition to the di-block FG nups, which implies that the DCBG model which originally included only the di-block FG nups could be made more biologically relevant by including the single-block FG too. Deletion experiments on single-block FG nups have also revealed that they are critical for proper functioning of the NPC in Baker’s yeast [31, 32]. In this paper we attempt to understand what role single-block FG nups may play when introduced to the DCBG model, Fig 1B, which we refer to as our modified DCBG model.

Results from our molecular simulations demonstrate that single-block FG nups are compact and therefore localized to their anchor points within the NPC. These anchor points are located on the inner wall of the NPC [27]. Through polymer brush modeling we find that adding a layer of single-block FG nups to the DCBG model increases the range of cargo sizes which are able to translocate the pore via a cooperative interaction with the tips of di-block FG nups. This suggests a functional reason for the presence of both single-block and di-block FG nups *in vivo*, with the combination increasing the diameter of cargo which can translocate the pore. This cooperative mechanism also provides a framework for enhancing polymer brush based pore transport in biomimetic applications.

## Materials and Methods

We first examined the polymer conformations adopted by single-block FG nups using molecular dynamics (MD) simulations. Given the large size of disordered of FG nups and the long timescales involved in their dynamics, coarse grained molecular dynamics simulations were utilized. The coarse grained (CG) model by Hills *et al.* [33] was used, which is detailed enough that it is able to accurately reproduce the secondary and tertiary structure of proteins, and maintains the ability to fold small proteins correctly *ab initio* starting from fully extended states. An implicit solvent is used resulting in rapid simulation speed and which allows for the capability of simulating multi-protein systems over microsecond timescales [11]. The Hills model reproduces the energy landscape of proteins as found *in vivo* by representing each amino acid by multiple CG bead interaction sites and by using specially developed interactions between these sites. The interactions between the CG beads in the Hills model are determined by measuring the forces present in highly accurate all atom simulations of diverse protein sequences to create a CG interaction following the multiscale coarse-graining (MS-CG) method [34]. This multiscale method makes no a priori assumptions about the form of interactions between CG beads, and implicitly includes multibody correlations [35] in the resulting effective CG potentials. The secondary and tertiary structure of proteins emerges naturally in the CG simulations through the comprehensiveness of the physical model, with the emergent CG model interactions general enough to simulate proteins of arbitrary amino acid sequence.

CG models typically require a normalization step where CG potentials for a specific protein simulation are scaled such that model results match well with experiment [33, 36]. In a previous publication [11] we found a scaling factor for the Hills model which reproduces the biphasicness of FG nups as seen in experimental results obtained by Yamada *et al* [30] for di-block type FG nups in *S. cerevisiae*. The experiments themselves indicated that many FG nups are individually di-blocks, with one domain resembling an extended polymer and non-interacting in general while FG domains are collapsed coils and cohesive to other FG domains. Our previously determined simulation scaling factor [11] reproduces the biphasicness of FG nups in terms of both cohesion and polymer structure. In this manuscript we utilize the same scaling factor as determined previously to ensure that simulations of single-block type FG nups from *S. cerevisiae*, consisting of Nup42, Nup49, and Nup57, are consistent with the experimental results on di-block FG nups by Yamada *et al* [30]. The amino acid regions of the FG nups simulated and domain definitions used are also from Yamada *et al* [30], therefore our simulations of single-block FG nups can be directly compared to their experimental characterizations.

The CG Hills *et al* [33] model simulations were run in the large-scale atomic/molecular massively parallel simulator (LAMMPS) [37] software package. Using a scaling factor of 4.30 at 300 K, simulated single-block FG nups from *S. cerevisiae* were equilibrated for 1 microsecond, using starting conditions which consisted of fully extended protein chains. Disordered regions of FG nups which were simulated used the nup specific definitions defined in Yamada *et al* [30]. After equilibration, data was then taken from a 4 microsecond production run on which post simulation analysis was performed to determine the average radius of gyration of different domains as defined in Yamada *et al* [30]. Contact proximity between two given side-chain beads was calculated by counting the number of times side-chains were within 16 Angstroms of each other in the trajectory snapshots which were saved every 100 picoseconds during the 4 microseconds of production simulation, with contact number normalized by the number of snapshots to determine the contact probability. The sequences for the FG nups simulated are given in S1 Appendix.

## Results

### Coarse Grained Simulations

**Fig 3:**
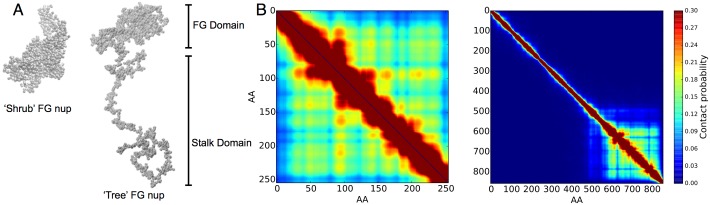
Coarse grained molecular modeling of single-block FG nups. A: Left is a simulation snapshot of full length Nup57 showing the single polymer block FG domain. Right shows a snapshot of full length Nup1 demonstrating the di-block structure of FG and extended domain structures. B: Left, the contact probability map for Nup57 show the time-averaged contacts between all pairs of amino acids. A monolithic block diagonal structure fills the entire contact map, implying that this FG nup is comprised of a single polymer block FG domain. Right, in contrast, the contact map for human Nup1 shows one block for the FG domain and diagonal contacts for the extended polymer domain, with both domains having low probability of cross contact.

We find that the single-block FG nups simulated in this paper have a single block amino acid sequence structure which can be described as a single FG domain, corresponding to a continuous sequence region with a low concentration of charged amino acids and a high concentration of FG motifs [38]. Polypeptide chains representing the disordered regions of these FG nups were simulated from an initially fully extended configuration and equilibrated before the start of simulation for 1 *μ*s, see S1 Appendix for simulation convergence data during the equilibration period. Fig 3A shows a typical snapshot of one such nup, Nup57, after collapse and equilibration. This snapshot indicates that single-block FG nups tend to exhibit a single block polymeric structure which resembles a collapsed coil structure. Nup42, Nup49, and Nup57 had *R*_*g*_ values of 2.4 nm, 2.0 nm, and 2.0 nm respectively over 4 *μ*s of simulation which is consistent with these FG nups being collapsed coils.

**Fig 4:**
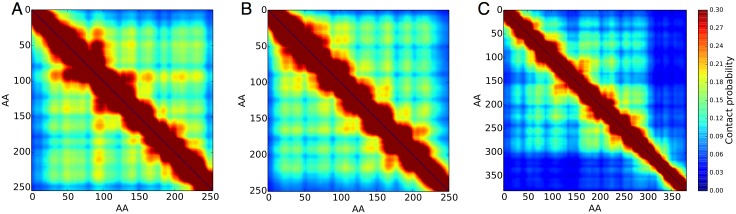
Contact maps for the different single-block FG nups in Baker’s yeast from coarse grained molecular modeling. A: Nup57’s contact map B: Nup49’s contact map C: Nup42’s contact map. Contact probability maps show the time averaged contacts between all pairs of amino acids. A block diagonal structure in the contact maps represents a collapsed coil structure, which describes the single-block FG nups well, except for a small region of around 80 amino acids in length where Nup42 anchors to the pore wall. Amino acid residues shown are with respect to the disordered domains of the simulated FG nups, while full protein amino indexes can be determined by domain definitions in Yamada *et al* [30].

To characterize the polymeric structure of individual FG nups, we defined the contact probability between a pair of amino acids as the percentage of time the corresponding CG backbone beads are within 16 Angstroms of each other. This contact probability is plotted as probability maps over all possible amino-acid contact pairs, Figs 3B and 4. Generally, these contact maps show a monolithic block diagonal structure for the single-block FG nups that represents a collapsed disordered structure, except for a very short extended domain where Nup42 is anchored to the NPC pore wall. For all simulated nups, the contact maps indicated that the individual FG nups were highly dynamic and disordered. This can be seen in the relatively smooth block contact structure of simulated nups, with contacts between amino acids rapidly exchanging and no folded constant contacts visible. The contact maps of our simulated single-block FG nups differ significantly from previously reported contact maps of di-block FG nups (Fig 3B) [11]. Single-block FG contact maps generally lack an extended disordered domain which predominately avoids contact with itself and FG domains. Here, our simulation results on the full disordered regions of single-block FG nups support the hypothesis that FG nups exists in a bimodal configuration [30] by demonstrating that it is clearly possible that individual FG nups can form “shrub” like single-block polymers rather than “tree” like di-block structures which have been reported earlier [30].

### 0.1 Polymer brush morphology and dynamics with single-block FG nups

**Fig 5:**
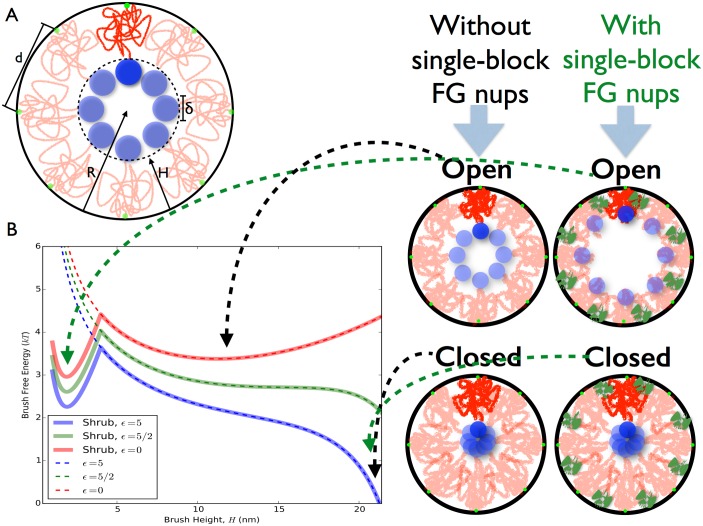
Mean field polymer brush model of the Nuclear Pore Complex. A: Schematic of a polymer brush structure formed by di-block FG nups. Parameters *H*, height of the brush; *R*, radius of the pore; *δ*, diameter of the “sticky tips”; and *d*, the average distance between anchor points. Green circles represent the locations at which FG nups are grafted to the pore. B: Free energy of the Nsp1 brush, with and without single-block FG nups present on the pore wall, as a function of brush height for various values of the blob cohesive energy (*ϵk*_*B*_*T*). Brush height can extend to a maximum of around 22 nm, which is the radius *R* of the modeled pore minus the size of the sticky tips. Di-block FG nup tip to single-block FG nup cohesion is fixed at *ϵ*_*s*_ = 6*kT*. Right: Schematic diagram of the proposed Di-block Copolymer Brush Gate model at various minima of the brush free energy. When particular transport factors are present which are able to outcompete the inter-FG domain “sticky tip” interactions, the brush is able to open up to a new free energy minimum that can accommodate the cargo. When interactions between sticky tips are able to recover into the several *kT* range, the pore is able to close with a free energy minimum at *H* ∼ *R* − *δ*. We have previously estimated the self interaction energy level of the Nsp1 sticky tip to be 4.7 *kT* [11], which also sets the energy scale for blob-blob interactions of *ϵkT*. Single-block FG nups (shrubs) denoted by green compact polymers in the schematic, produce a new minimum in the free energy close to the wall, thus potentially allowing for wide-open states of the brush if tip-tip cohesiveness is lost or reduced.

We turn to polymer physics modeling to understand the underlying biophysical mechanisms that may govern the formation and dynamics of different types of mesoscale structures within the NPC which play a role in transport regulation. First, we summarize a simple polymer brush model, the *Di-block Copolymer Brush Gate* (DCBG) model [11], that has been successfully used to model aggregates of di-block type FG nups. This model treats the core of the NPC as an idealized cylindrical polymer brush constructed of di-block polymers. It is assumed that the extended domain region of the nups can be described by neutral excluded volume polymers with an appropriate length and stiffness, and that they are grafted to the inside of a cylinder that is geometrically representative of the nuclear pore. The biphasic structure of these gating di-block FG nups is incorporated by modeling the ends of the polymers, the FG domains, as structureless cohesive blobs that correspond to the cohesive FG domains at the FG nup tips. The resulting configuration is a polymer brush as shown schematically in Figs 1A and 5. Here we extend this model to include the presence of single-block FG nups around the pore wall, which are present in Baker’s yeast, Fig 1B [30].

We consider a polymer brush confined to the inside of a cylinder of radius *R*, with di-block FG nups modeled as chains of length *N* monomers of Kuhn length *a* with sticky tips of size *δ*. The chains are separated at their anchor points along the wall by a grafting distance *d*, see Fig 5. An equal number of single-block FG nups were modeled as sticky blobs of size *δ*_*s*_ grafted along the pore wall with the same grafting distance. In a modified DCBG model with single-block FG nups present we can solve for the height *H* of the resulting brush via polymer brush modeling using scaling theory. The height of this polymer brush at equilibrium can be derived via a minimization of the free energy of the brush which has contributions coming from (i) entropic stretching of the extended domains (ii) the excluded volume interactions (steric hindrance) between the extended domains (iii) the cohesive energy interactions between the tip blobs and (iv) the cohesive energy interactions between tip blobs and single-block FG nups. The modified DCBG model includes an extra term (iv) which accounts for the attraction of the single-block FG nups with the tips of di-block FG nups. The DCBG model and its single-block FG nup extension, which are both models of polymer brushes over a negatively curved surface with cohesive tips coupled to the free ends of the polymers, result in a brush height which is given by the solution to a non-linear differential equation. An analytical solution however can be arrived at by making a mean-field approximation, which results in an algebraic form for the effective free energy per chain [11], as per Eq 1;

$$\begin{array}{c} \frac{F}{k_{B}T}∼{\left(H/a\right)}^{5/2}N^{-3/2}+\frac{a^{5/2}N^{3/2}RH^{-1/2}}{d^{2}\left(2R-H\right)}-\frac{\epsilon \delta^{2}R}{d^{2}\left(R-H\right)}-\frac{\epsilon_{s}\delta^{2}\left(\delta_{s}-H\right)R\Theta \left(\delta_{s}-H\right)}{d^{2}\delta_{s}R_{s}}, \end{array}$$ (1)

The Heaviside step function Θ(*x*) is defined to be 1 if *x* is positive and 0 if *x* is negative, with *ϵk*_*B*_*T* and *ϵ*_*s*_*k*_*B*_*T* equal to the effective cohesive energy between a pair of tip blobs and between single-block FG nups and di-block FG nups tip blobs respectively. The first two terms of the free energy follow directly from prior work on polymer brushes in cylinders [39, 40], which treats the cylindrical brush as being locally an Alexander—de Gennes brush. More recent models fix deficiencies and problematic predictions of the Sevick model [41], yet we keep the older Sevick model to maintain similar levels of rough physical approximation for different brush interactions. The Kuhn length *a* and the effective polymerization number *N* in the brush free energy have been measured to be twice the monomer length of amino acids in the chain and half the number of amino acids in the disordered protein chains, respectively [11]. *R*_*s*_ is the radial distance at which the single-block FG nups are located at within the pore. The cohesive domains at the tip are in a collapsed state as measured from CG modeling [11], with the sticky tip blobs of size (diameter) *δ* = 3.6 nm, twice the radius of gyration as measured previously via CG model simulations [11]. We make the assumption that the average spacing between grafting points of a given di-block FG nup, for example Nsp1, along the pore wall is *d* ∼ 10 nm, which we estimate by assuming that there are 32 copies [3] of Nsp1 anchored along the inner wall in a symmetric fashion along the geometry used in Yamada et al [30]. The pore radius is taken to be *R* ∼ 25 nm [30]. For the single-block FG nups size *δ*_*s*_ we use a value of 4 nm which is roughly twice the average radius of gyration of collapsed FG domains of single-block FG nups Nup42, Nup49, and Nup57 which are relatively similar in size. The location of the single-block FG nups are not precisely known, with *R*_*s*_ = 25 nm in scenarios where the single-block FG nups are located along the pore wall [27]. The third term in the brush free energy assumes a uniform random mixing of all sticky tip blobs with each other along a doughnut topology shaped cylindrical region along the center of the pore S1 Appendix. Although this assumption breaks down when the pore opens widely, tip-tip interactions are at their lowest point in such a scenario, and absolute free energy approximation should remain roughly consistent. Term (iv), the cohesive energy interaction between tip blobs and single-block FG nups was derived from the probability of single-block FG nup and tip contact, which equals the product of tip and single-block FG nup concentrations within the pore as they spatially overlap, while the cohesive energy interactions between the tip blobs was similarly derived as a tip to tip contact probability, equal to the square of the tip blob concentration as derived (see S1 Appendix).

Fig 5 shows the free energy of the modified DCBG model as a function of the brush height for three different values of the tip-tip cohesive energy and a fixed tip to single-block FG nup cohesiveness of *ϵ*_*s*_ = 6*kT*. For a given tip-tip cohesive energy, the equilibrium value of the brush height is located at the minimum of the free energy profile. When there is no tip cohesion, corresponding to situation where there is complete screening of tip-tip interactions by transport factors and *ϵ* = 0, we can see that, in the absence of single block FG nups, the equilibrium brush height is lowered to less than at least half the pore radius, which implies that if cohesion between the tips is lost a wide-open channel down the center of the pore can be formed for cargo to travel through. We term this the open state of the gate. If the single block FG nups are present, we note that there is a new minimum for brush heights close to the NPC wall which then becomes the “open” state in this case. Here the wide open state of the brush is stabilized by the attractive interactions between the single block FG nups and the sticky tips of the diblock FG nups. As the cohesive energy is increased, we see a new minimum developing at *H* ∼ *R* − *δ*, which is a state where the extended domains have stretched to such a degree that the cohesive tips are at the center of the pore, completely closing the conduit. We term this as the closed state of the pore. This indicates that there can be a transition between the closed and open states of the polymer brush as the tip-tip cohesion energy is varied. Thus the arrangement of individually biphasic functional polymers, with an extended domain and collapsed cohesive tip, leads to a brush structure that can switch between open and closed states, as shown in Fig 5, with relatively small changes in the cohesiveness of polymer tips. Importantly, the free energy of the brush indicates that the nups extend all the way into the center of the pore when transport factors are not present and tip-tip cohesion is high, while the FG nups form a brush with an intermediate height that leaves an open transport conduit for transport along the middle of the pore when tip-tip cohesion is low.

With regards to the function of gated NPC transport *in vivo*, it is known that transport factors, such as karyopherins and exportins, bind to FG motifs [42, 43]. This provides a mechanism which can screen tip-tip interactions, lowering *ϵ* via the disruption of the inter-molecular cohesiveness between nup tips by the competitive binding of transport factors. We propose that the binding of transport factors *in vivo* results in such a switch from the closed to open state forming the basis for our DCBG model. Specific types of transport mediated cargo complexes, which have an attraction to di-block FG nup tips, could therefore have the ability to dissolve into the central plug that keeps the gate closed, thus effectively opening it up to the desired diameter. Once open, these cargo complexes can then undergo one dimensional diffusion down the center of the NPC channel, until enzymes on the nuclear side cleave the transport factors which reduces the propensity of cargo to remain in the channel leading to a rapid exit to the nucleus. For export, this picture would be reversed with cargos starting in the nucleus and cleaving enzymes located on the cytoplasmic side of the NPC.

**Fig 6:**
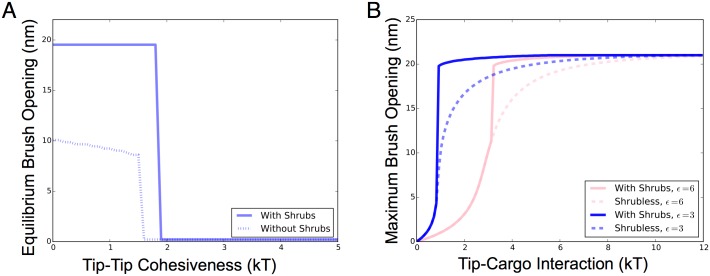
Overview of brush response to tip-tip and tip-transport factor interaction levels in the Di-block Copolymer Brush Gate (DCBG) model with single-block FG nups. A: Changes in tip-tip cohesiveness result in equilibrium brush openings that have the nuclear pore in either significantly open or completely closed states, with a sharp transition at ∼1.6 kT when single-block FG nups (shrubs) are not present and ∼1.9 kT when single-block FG nups are present. The single-block FG nups almost double the equilibrium brush opening for weak tip-tip cohesiveness below ∼1.6 kT. Tip to single-block FG nup cohesion is fixed at *ϵ*_*s*_ = 6*kT*. B: Maximum brush opening assuming the energy of tip-cargo interaction is added to the brush free energy, resulting in an increase in the possible opening of the pore. Maximum brush opening is plotted for different amounts of free energy introduced into the brush system by tip-transport factor binding for two different values of the tip to tip cohesion *ϵ*. Tip to single-block FG nup cohesion is fixed at *ϵ*_*s*_ = 6*kT*.

From the free energy of the DCBG and its modified model, we clearly see that the presence of single-block FG nups substantially increases the equilibrium opening of the NPC, Fig 6A. Thus, the presence of single-block FG nups allows the pore to open to a considerably greater extent. We also used polymer brush modeling to understand in detail how the strength of the effective tip-tip cohesion, which is modulated by the presence of transport factors, changes brush structure and its receptivity to transport, Fig 6A. We find that tip-tip cohesiveness above ∼1.9 kT (when single-block FG nups are present) provides for a critical level of tip cohesion which allows the pore to be in a closed state. Lower tip cohesiveness results in a rapid transition in brush structure, with the brush opening up to 40 nm in diameter below the critical tip cohesiveness. This rapid change in brush structure as a function of tip cohesion is a result of the relatively flat free energy of the brush system, Fig 5, where large changes in brush height can have small differences in the brush free energy. This is likely to be functionally useful in designing generalized “spring-loaded” polymer gating pores.

Similar to how transport factors and cargo screen tip-tip interactions to modulate tip-tip cohesiveness, transport factors also introduce a free energy of binding, from transport factors to tips, into the pore brush system. Given the free energy profile of the brush, an increase in brush free energy typically results in a lower possible brush height and further opening of the pore if cargo are present, Fig 5. The maximum amount of brush opening assuming tip-cargo free energy is added to the brush free energy is plotted in Fig 6B. For any given fixed tip-tip interaction, there exists a sharp transition from closed to open brush states as the transport factor-tip binding energy as increased, beyond which the return from increasing the binding energy further results in negligible increased pore opening. Again, this is a direct result of the relatively flat free energy curve in the absence of the single block FG nups. In the presence of the single block nups there is a sharp first-order transition in the brush opening because of the new minimum in the brush free energy introduced for small brush heights. For values of *ϵ* ∼ 3 *kT* the maximum brush opening of the pore can be up to four times greater, Fig 6B, if single-block FG nups are present. The localization of single-block FG nups along the pore wall therefore results in a transition from closed to open brush states at lower free energies in both tip-tip cohesion and tip-cargo interactions as well as much greater magnitudes in the size of the opening.

## Discussion

The mechanisms behind NPC transport regulation remain poorly understood, with many experimental results regarding the basic properties of individual FG nups, such as their structure, location and cohesive properties, in conflict. The aggregate properties of these FG nups when put together within the confines of the NPC channel are even more uncertain. Here we have attempted to understand how the overall architecture of the assembly of both “tree” type di-block FG nups and “shrub” type single-block FG nups in the NPC channel is governed by the properties of the individual FG-nups and their physical polymer properties, rather than just studying the di-block FG nups alone as has been done before. Using a model where individual FG nups can have specific domains which are compact or extended, our results indicate that a Forest type model structure [30] emerges, with the “sticky tips” of di-block FG nups coalescing in the center of the pore to form a hydrophobic plug, which is connected by an extended coil brush zone to the pore wall where single-block FG nups are localized. This three part structure results in a spring loaded polymer brush gate which is capable of opening for very large cargo. Indeed, recent high speed AFM imaging of nuclear pores [44] shows that some pores spend a significant amount of time in a “radial” arrangement, where there exits an amalgamation of FG nup tips at the center of the pore supported by dynamic “stalk” regions.

In this paper we have focused on the effects of adding single-block FG nups to the larger di-block FG nups, both of which have been shown to be critical for many forms of transport [31] in Baker’s yeast. Some details of NPC structure such as cytoplasmic filaments and nuclear basket structure have not been considered in our analysis. Since the cytoplasmic filaments and the basket are spatially well separated from the interior of the pore we anticipate that they would not interfere with the copolymer brush structure. Given that the properties of the single-block FG nups are similar to the sticky tip FG domains of di-block FG nups in terms of bimodal adhesion [30], we show that the single-block FG nups interactions with di-block FG nup tips helps to stabilize the wide open configurations of the copolymer brush in the presence of large cargo in the middle of the pore. The sort of cooperativity between single-block and di-block FG nups that we observe in terms of opening the pore to a wider degree could be relevant for the export of large ribonucleoprotein particles whose transport is facilitated by surface bound transport factors. In the work of Strawn *et al* [31] single-block FG nups like Nup49 or Nup57, together with di-block FG nups like Nup100 or Nup116, were required for the viability of yeast cells, yet the deletions of any one of these nups by themselves resulted in viable cells. Given that the formation of the NPC’s permeability barrier is the most fundamental function of the NPC, this deletion data likely indicates that the presence of both classes of FG nups are required for the NPC in Baker’s yeast to spatially form a permeability barrier across the pore using a central plug along the core and “shrub” FG nups along the wall. Additionally, deletions of just Nup49 or Nup57 resulted in only negative perturbations to large cargo mRNA export, while deletions of di-block FG nups only had no effect on mRNA export [32], indicating that single-block FG nups are likely necessary for the transport of large cargoes via cooperativity with the sticky tips of di-block FG nups. Our modified DCBG model can explain both the requirement for having both single-block FG nups and di-block FG nups for viability and the necessity of having a critical number of single-block FG nups present for the transport of large cargos such a mRNA.

We have studied the equilibrium structure of the NPC brush for various tip-transport factor interaction energies Fig 6B. Given that the FG nup tip-tip interaction energies and the magnitude of screening by transport factors have not been studied experimentally, we can not immediately calculate the resultant optimal tip-transport factor interaction energies for brush opening, although we can outline the process. Qualitatively we find that as the level of tip-tip screening by transport factors increases, the free energy gained from tip-transport factor binding that is required to open the pore decreases strongly. Given that it has been observed that rapid cargo transport through the NPC is likely due to rapid exchange of transport factor-tip partners as the cargo moves through the pore [45], a high off rate equivalent to the inverse of the tip-tip exchange timescale will be advantageous. A high off rate such as this necessarily implies that the tip-transport factor binding energy will be small. With a small tip-transport factor binding energy, opening of the pore must rely more on transport factor screening of the di-block FG nup tips rather than thorough the free energy change of the brush through tip-transport factor binding. The optimal properties of transport factors are therefore likely to involve two factors, low tip binding energies for a high off rate and a high propensity for tip screening via geometric or competitive binding effects [46]. Additionally, maximizing the total cargo flux likely involves simultaneously opening the pore around as many cargoes as possible while maximizing the transport factor off rate. We find that both increased tip-tip screening or increased tip-cargo binding energies can achieve increased brush opening (Fig 6), although increased tip-tip screening by transport factors is more desirable as the off rate is less likely to be reduced, resulting in more rapid transport. These conclusions should hold generally true given that our assumptions for the brush free energy scaling are reasonable and that the extended regions of the FG nups studied are disordered proteins which can be studied by mean field polymer theory. Some further limitations to our modeling are that we do not address the kinetics and dynamic behavior of the polymer system, although this is planned for a future study, and we currently ignore the effect of counterions in solution on the charged extended “stalk conformations” of tree FG nups, although these counterion effects at the level of polymer scaling theory can be considered effectively as just modifying stalk sizes in the model.

## Conclusion

Our results not only provide for a consistent physical mechanism by which we can understand nucleocytoplasmic transport and the puzzling presence of “shrub” type single-block FG nups, but our model is also very much applicable to designing and optimizing novel forms of biomimetic transport. The application of biomimetic membranes [47] has a wide variety of uses ranging from chemical and biological separation to purification [48]. Additionally these membranes provide a platform for the analytical detection of substances, drug delivery [49], as well as for self-contained reactors and mock cells [50, 51]. The regulated cross-membrane trafficking of cargos through the membranes in such systems is of high importance and our investigations of a relatively simple biologically compatible mechanism could be very useful. In general, any di-block polymers with the extended domain near the polymer grafting point and a collapsed and cohesive tip domain could be used to design biomimetic pores which regulate traffic using our DCBG mechanism. Similar to our modified DCBG model, cohesive collapsed coil polymers can be added along the biomimetic pore wall to increase the size of cargos which can transport the pore. As evidenced by early efforts to make biomimetic gates inspired by the NPC [52, 53], our DCBG models should find many opportunities for implementation outside of the NPC context from which they are inspired.

## Supporting Information

S1 Appendix Supporting Calculations and Data. Includes (1) a derivation of the NPC cylindrical polymer brush total free energy per chain, (2) demonstration of coarse grain FG nup simulation convergence, and(3) the studied FG nup sequence information.
(PDF)
