# Supplementary material for: Cooperative Interactions between Different Classes of Disordered Proteins Play a Functional Role in the Nuclear Pore Complex of Baker’s Yeast
Source: PLoS One. 2017 Jan 9;12(1):e0169455. doi: 10.1371/journal.pone.0169455 (PMC5222603; doi:10.1371/journal.pone.0169455)
Supplement: S1 Appendix — Includes (1) a derivation of the NPC cylindrical polymer brush total free energy per chain, (2) demonstration of coarse grain FG nup simulation convergence, and(3) the studied FG nup sequence information. (PDF) [file pone.0169455.s001.pdf]

Supporting Material

Cylindrical Brush Free Energy

Characterization of the NPC cylindrical polymer brush is achieved by deriving its total free energy per chain:

$$F_T = F_s + F_{ex} + F_{total_{coh}} \tag{1}$$

The first term in the RHS of this equation, F_s, is the stretching free energy which we model as originating from stalk part of the NPC brush only. The second term F_{ex} is the excluded volume free energy of this cylindrical brush, and the last term is the total cohesive energy $F_{total_{coh}}$ resulting from the sticky tip FG domains.

1 Free energy of stalk chain stretching

The per chain stretching free energy is (1):

$$F_s = k_b T(\#blobs_{stalk}) = k_b T \int_{stalk} \mathrm{d}n_b \tag{2}$$

With n_b the number of blobs per nup and where the stretching free energy per nup is equal to Boltzmann's constant times the temperature times the number of blobs in the stalk region of a nup.

The number of blobs in the stalk brush if we have a *flat brush* is:

$$n_b = L/\xi \tag{3}$$

With L the end to end distance of stalk chains and ξ the radius of the stalk brush blobs.

The number of monomers per extended chain is:

$$N = n_b(\xi/a)^{5/3} \tag{4}$$

With a equal to the length of monomers in the chains.

$$\Rightarrow N = (L/\xi)(\xi/a)^{5/3} \tag{5}$$

$$\Rightarrow \xi = (N/L)^{3/2} a^{5/2} \tag{6}$$

Now we can consider the cylindrical brush case. First we consider blob size changes along the stalk brush as a function of the contour length s, which is a function of monomer number m. i.e. m is the mth

monomer starting from the direction of the center of the cylinder with $m = 0$ the free end of the stalk chain: In direct analogue to the flat brush case, Eq. 6:

$$\Rightarrow \xi(s) = (\frac{\mathrm{d}m}{\mathrm{d}s})^{3/2} a^{5/2} \tag{7}$$

We can now calculate the change in the number of blobs as a function of s, analogous to Eq. 3, as:

$$\Rightarrow \mathrm{d}n_b = \frac{\mathrm{d}s}{\xi(s)} \tag{8}$$

Which after substitution from Eq. 18 becomes (2):

$$\Rightarrow \mathrm{d}n_b = \mathrm{d}s(\frac{\mathrm{d}s}{\mathrm{d}m})^{3/2} a^{-5/2} \tag{9}$$

We can now solve for the stretching free energy in Eq. 2

$$F_s = k_b T \int_{stalk} \mathrm{d}n_b = N_c k_b T \int \mathrm{d}s(\frac{\mathrm{d}s}{\mathrm{d}m})^{3/2} a^{-5/2} \tag{10}$$

$$= k_b T \int \mathrm{d}m(\frac{\mathrm{d}s}{\mathrm{d}m})(\frac{\mathrm{d}s}{\mathrm{d}m})^{3/2} a^{-5/2} \tag{11}$$

$$= k_b T \int \mathrm{d}m(\frac{\mathrm{d}s}{\mathrm{d}m})^{5/2} a^{-5/2} \tag{12}$$

Which after the mean field approximation of:

$$\frac{\mathrm{d}s}{\mathrm{d}m} = \frac{H}{N} \tag{13}$$

With H the height of the cylindrical stalk brush. Eq. 12 reduces to:

$$F_s = k_b T N (\frac{H}{N})^{5/2} a^{-5/2} \tag{14}$$

Where we can now conclude that the stretching free energy per chain is:

$$F_s = k_b T (\frac{(H/a)^{5/2}}{N^{3/2}}) \tag{15}$$

2 Free energy of excluded volume interactions

For a flat brush the free energy per chain of excluded volume interactions is (1):

$$F_e = \frac{1}{2} k_b T (\#blobs_{stalk}) \rho_{blob} \tag{16}$$

With ρ_{blob} the volume fraction of blobs in the brush.

This can be generalized to a per chain free energy valid for cylindrical brushes:

$$F_s = \frac{1}{2} k_b T \int_{stalk} \mathrm{d}n_b \rho_{blob} \tag{17}$$

With n_b the number of blobs per nup. The local volume faction can be defined as:

$$\rho_{blob} = \frac{\mathrm{d}V_{blobs}}{\mathrm{d}V_{brush}} = \frac{\xi^3 N_c \mathrm{d}n_b}{2\pi s \mathrm{d}s L} \tag{18}$$

Which implies that:

$$F_s = \frac{1}{2} k_b T \int_{stalk} \mathrm{d}n_b \frac{\xi^3 N_c \mathrm{d}n_b}{2\pi s \mathrm{d}s L} \tag{19}$$

Which simplifies to:

$$F_s = \frac{1}{2} k_b T \int_{stalk} \mathrm{d}s \frac{\mathrm{d}n_b^2}{\mathrm{d}s^2} \frac{\xi^3 N_c}{2\pi s L} \tag{20}$$

Which after substitution for $\frac{\mathrm{d}n_b}{\mathrm{d}s}$ by Eq. 8 equals:

$$F_s = \frac{1}{2} k_b T \int_{stalk} \mathrm{d}s \frac{\xi N_c}{2\pi s L} \tag{21}$$

Which after substitution for ξ from Eq. 18:

$$F_s = \frac{1}{2} k_b T \int_{stalk} \mathrm{d}s \frac{(\frac{\mathrm{d}m}{\mathrm{d}s})^{3/2} a^{5/2} N_c}{2\pi s L} \tag{22}$$

Which equals:

$$F_s = \frac{1}{2} k_b T \int_{stalk} \mathrm{d}m \frac{\mathrm{d}s}{\mathrm{d}m} \frac{(\frac{\mathrm{d}s}{\mathrm{d}m})^{-3/2} a^{5/2} N_c}{2\pi s L} = k_b T \int_{stalk} \mathrm{d}m \frac{(\frac{\mathrm{d}s}{\mathrm{d}m})^{-1/2} a^{5/2} N_c}{\pi s L} \tag{23}$$

Which after the mean field approximation of (2):

$$\frac{\mathrm{d}s}{\mathrm{d}m} = \frac{H}{N} \tag{24}$$

and

$$s = R - \frac{H}{2} \tag{25}$$

becomes:

$$F_s = k_b T \int_{stalk} \mathrm{d}m \frac{(\frac{H}{N})^{-1/2} a^{5/2} N_c}{(2R - H)L} = k_b T N \frac{(\frac{H}{N})^{-1/2} a^{5/2} N_c}{(2R - H)L} = k_b T N^{3/2} \frac{H^{-1/2} a^{5/2} N_c}{(2R - H)L} \tag{26}$$

Which after substitution for the number of chains in the brush N_c:

$$N_c = RL/d^2 \tag{27}$$

leads to a per chain excluded volume free energy of:

$$F_{ex} = k_b T (\frac{N^{3/2} a^{5/2}}{d^2}) \frac{R H^{-1/2}}{2R - H} \tag{28}$$

3 Free energy of tip-tip cohesive interactions, f_{coh}

We define f_{coh} to be the absolute value of the energy density of sticky tip interactions, representing part of the $F_{total_{coh}}$ term. Similar to the flat brush case, for the sticky tip to sticky tip interactions we can define an energy density of blob interactions to have an energy of $\epsilon k_b T$:

$$f_{coh} = 2\epsilon k_b T c^2 V \tag{29}$$

We have $c = N_c N_b / V$ the concentration of blobs (for N_b the total number of blobs per chain). For each chain or FG nup there exists only one sticky tip, therefore N_b =1 in this case. The volume the sticky tips can take on is approximated as an extended cylindrical region atop the stalk brush region whose volume is $V = 2\pi(R - H)L\delta$, with L equal to the height of the brush region axially along the pore.

N_c is the number of chains determined by the grafting distance d, with

$$N_c = 2\pi RL/d^2 \tag{30}$$

The concentration c is therefore:

$$c = \frac{R}{d^2\delta(R - H)} \tag{31}$$

Which results in a free energy density of blob interactions of:

$$f_{coh} = 2\epsilon k_b T c^2 V \sim \epsilon k_b T \frac{R^2}{d^4\delta^2(R - H)^2}(R - H)L\delta \tag{32}$$

Which can be simplified to:

$$f_{coh} = \epsilon k_b T \frac{R}{d^2\delta(R - H)} N_c \tag{33}$$

The total cohesive energy *per chain* is then equal to the volume of a sticky tip blob times the cohesive free energy density of all blob interactions divided by the number of chains.

$$F_{coh} = \epsilon k_b T \frac{R}{d^2\delta(R - H)} N_c \delta^3 / N_c = \epsilon k_b T \frac{R\delta^2}{d^2(R - H)} \tag{34}$$

4 Free energy of tip-shrub cohesive interactions, f_{shrub}

We define f_{shrub} to be the absolute value of the energy density of sticky tip to shrub interactions, representing part of the $F_{total_{coh}}$ term. Similar to the tip-tip free energy we can define an energy density of shrub blob to tip blob interactions given that blob-blob interactions have an energy of $\epsilon_s k_b T$:

$$f_{shrub} = 2\epsilon_s k_b T c_t c_s V_o \tag{35}$$

Where V_o is the overlap volume of the different types of blobs.

For δ equal to the blob size of sticky tips, we have c_t from Equ. 31:

$$c_t = \frac{R}{d^2\delta(R - H)} \tag{36}$$

We have $c_s = N_c N_b / V$ the concentration of shrub blobs (for N_b the total number of blobs per chain). For each chain or FG nup there exists only one shrub, therefore N_b =1 in this case. The volume the shrubs can take on is approximated as a hallowed extended cylindrical region extending from the wall of the pore to radius R_s whose volume is $V = 2\pi R_s L \delta_s$, with L equal to the height of the brush region axially along the pore.

N_c is the number of chains determined by the grafting distance d, with

$$N_c = 2\pi RL/d^2 \tag{37}$$

The concentration c_s is therefore:

$$c_s = \frac{R}{R_s d^2 \delta_s} \tag{38}$$

Which results in a free energy density of blob interactions of:

$$f_{shrub} = 2\epsilon_s k_b T c_t c_s V_o \sim \epsilon_s k_b T \frac{1}{d^2 \delta_s} \frac{R^2}{d^2 \delta (R-H) R_s} V_o \tag{39}$$

Which can be simplified to:

$$f_{shrub} = \epsilon_s k_b T \frac{V_o N_c R}{L d^2 \delta_s \delta (R-H) R_s} \tag{40}$$

The overlap volume is approximately $V_o = 2\pi(R-H)L(\delta_s - H)\Theta(\delta_s - H)$ for the Heaviside step function $\Theta(x)$ which is 1 if x is positive and 0 if x is negative. The free energy density is therefore:

$$f_{shrub} = \epsilon_s k_b T \frac{(\delta_s - H) N_c R \Theta(\delta_s - H)}{d^2 \delta_s \delta R_s} \tag{41}$$

The total cohesive energy *per chain* is then equal to the volume of a sticky tip blob times the cohesive free energy density of all blob interactions divided by the number of chains.

$$F_{shrub} = \epsilon_s k_b T \frac{(\delta_s - H) N_c R \Theta(\delta_s - H)}{d^2 \delta_s \delta R_s} \delta^3 / N_c = \epsilon_s k_b T \frac{\delta^2 (\delta_s - H) R \Theta(\delta_s - H)}{d^2 \delta_s R_s} \tag{42}$$

5 Free energy for the total brush

We can now solve for the total free energy of the cylindrical brush per chain:

$$F_T = F_s + F_{ex} - F_{coh} - F_{shrub} \tag{43}$$

$$F_T = k_b T (\frac{(H/a)^{5/2}}{N^{3/2}}) + k_b T (\frac{a^{5/2} N^{3/2}}{d^2}) \frac{R H^{-1/2}}{2R - H} - \epsilon k_b T \frac{R \delta^2}{d^2 (R-H)} - \epsilon_s k_b T \frac{\delta^2 (\delta_s - H) R \Theta(\delta_s - H)}{d^2 \delta_s R_s} \tag{44}$$

$$= k_b T \left(\frac{(H/a)^{5/2}}{N^{3/2}} + \frac{a^{5/2} N^{3/2}}{d^2} \frac{R H^{-1/2}}{2R - H} - \epsilon \frac{R \delta^2}{d^2 (R-H)} - \epsilon_s \frac{\delta^2 (\delta_s - H) R \Theta(\delta_s - H)}{d^2 \delta_s R_s} \right) \tag{45}$$

Simulation Equilibration

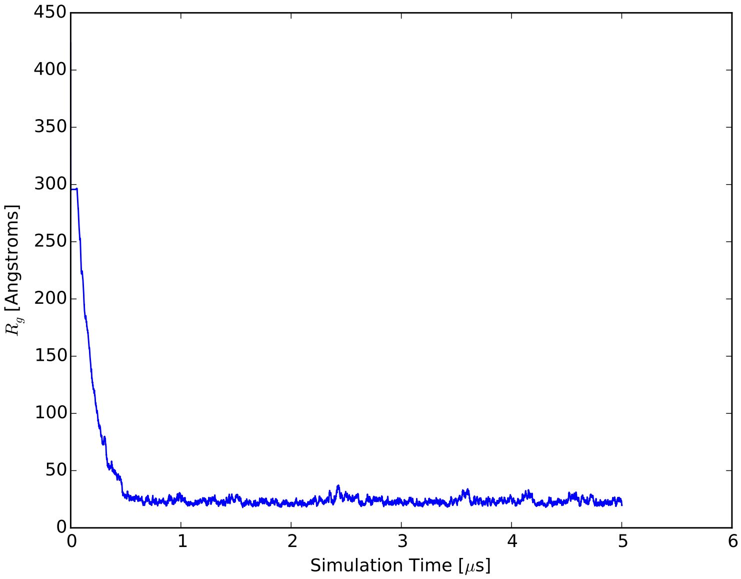

Figure 1: **Illustration of a CG FG nup simulation equilibration.** We use the first microsecond of our CG simulations as an equilibration period where data is not taken for characterization of FG nup properties. Only the last 4 microseconds of a CG simulation are considered the production run and have the molecular data analyzed. Shown is the radius of gyration for Nup42, starting as a fully extended polymer at time zero, which rapidly converges to a collapsed polymer structure after around 0.5 microseconds. Other FG nups which were simulated are of comparable size and converge on the same 0.5 microsecond timescale, which is well within the 1 microsecond initial equilibration time period.

FG nup sequences

Nup42: Amino Acids 1-382 were used.

MET SER ALA PHE GLY ASN PRO PHE THR SER GLY ALA LYS PRO ASN LEU SER ASN THR SER GLY ILE ASN PRO PHE THR ASN ASN ALA ALA SER THR ASN ASN MET GLY GLY SER ALA PHE GLY ARG PRO SER PHE GLY THR ALA ASN THR MET THR GLY GLY THR THR THR SER ALA PHE GLY MET PRO GLN PHE GLY THR ASN THR GLY ASN THR GLY ASN THR SER ILE SER ALA PHE GLY ASN THR SER ASN ALA ALA LYS PRO SER ALA PHE GLY ALA PRO ALA PHE GLY SER SER ALA PRO ILE ASN VAL ASN PRO PRO SER THR THR SER ALA PHE GLY ALA PRO SER PHE GLY SER THR GLY PHE GLY ALA MET ALA ALA THR SER ASN PRO PHE GLY LYS SER PRO GLY SER MET GLY SER ALA PHE GLY GLN PRO ALA PHE GLY ALA ASN LYS THR ALA ILE PRO SER SER SER VAL SER ASN SER ASN ASN SER ALA PHE GLY ALA ALA SER ASN THR PRO LEU THR THR THR SER PRO PHE GLY SER LEU GLN GLN ASN ALA SER GLN ASN ALA SER SER THR SER SER ALA PHE GLY LYS PRO THR PHE GLY ALA ALA THR ASN THR GLN SER PRO PHE GLY THR ILE GLN ASN THR SER THR SER SER GLY THR GLY VAL SER PRO PHE GLY THR PHE GLY THR ASN SER ASN ASN LYS SER PRO PHE SER ASN LEU GLN SER GLY ALA GLY ALA GLY SER SER PRO PHE GLY THR THR THR SER LYS ALA ASN ASN ASN ASN ASN VAL GLY SER SER ALA PHE GLY THR THR ASN ASN GLN SER PRO PHE SER GLY GLY SER GLY GLY THR PHE GLY SER ALA SER ASN LEU ASN LYS ASN THR ASN GLY ASN PHE GLN SER SER PHE GLY ASN LYS GLY PHE SER PHE GLY ILE THR PRO GLN ASN ASP ALA ASN LYS VAL SER GLN SER ASN PRO SER PHE GLY GLN THR MET PRO ASN THR ASP PRO ASN ILE SER LEU LYS SER ASN GLY ASN ALA THR SER PHE GLY PHE GLY GLN GLN GLN MET ASN ALA THR ASN VAL ASN ALA ASN THR ALA THR GLY LYS ILE

Nup49: Amino Acids 1-251 were used.

MET PHE GLY LEU ASN LYS ALA SER SER THR PRO ALA GLY GLY LEU PHE GLY GLN ALA SER GLY ALA SER THR GLY ASN ALA ASN THR GLY PHE SER PHE GLY GLY THR GLN THR GLY GLN ASN THR GLY PRO SER THR GLY GLY LEU PHE GLY ALA LYS PRO ALA GLY SER THR GLY GLY LEU GLY ALA SER PHE GLY GLN GLN GLN GLN GLN SER GLN THR ASN ALA PHE GLY GLY SER ALA THR THR GLY GLY GLY LEU PHE GLY ASN LYS PRO ASN ASN THR ALA ASN THR GLY GLY GLY LEU PHE GLY ALA ASN SER ASN SER ASN SER GLY SER LEU PHE GLY SER ASN ASN ALA GLN THR SER ARG GLY LEU PHE GLY ASN ASN ASN THR ASN ASN ILE ASN ASN SER SER SER GLY MET ASN ASN ALA SER ALA GLY LEU PHE GLY SER LYS PRO ALA GLY GLY THR SER LEU PHE GLY ASN THR SER THR SER SER ALA PRO ALA GLN ASN GLN GLY MET PHE GLY ALA LYS PRO ALA GLY THR SER LEU PHE GLY ASN ASN ALA GLY ASN THR THR THR GLY GLY GLY LEU PHE GLY SER LYS PRO THR GLY ALA THR SER LEU PHE GLY SER SER ASN ASN ASN ASN ASN ASN ASN ASN SER ASN ASN ILE MET SER ALA SER GLY GLY LEU PHE GLY ASN GLN GLN GLN GLN LEU GLN GLN GLN PRO GLN MET GLN CYS ALA

Nup57: Amino Acids 1-255 were used.

MET PHE GLY PHE SER GLY SER ASN ASN GLY PHE GLY ASN LYS PRO ALA GLY SER THR GLY PHE SER PHE GLY GLN ASN ASN ASN ASN THR ASN THR GLN PRO SER ALA SER GLY PHE GLY PHE GLY GLY SER GLN PRO ASN SER GLY THR ALA THR THR GLY GLY PHE GLY ALA ASN GLN ALA THR ASN THR PHE GLY SER ASN GLN GLN SER SER THR GLY GLY GLY LEU PHE GLY ASN LYS PRO ALA LEU GLY SER LEU GLY SER SER SER THR THR ALA SER GLY THR THR ALA THR GLY THR GLY LEU PHE GLY GLN GLN THR ALA GLN PRO GLN

GLN SER THR ILE GLY GLY GLY LEU PHE GLY ASN LYS PRO THR THR THR THR GLY GLY LEU PHE GLY ASN SER ALA GLN ASN ASN SER THR THR SER GLY GLY LEU PHE GLY ASN LYS VAL GLY SER THR GLY SER LEU MET GLY GLY ASN SER THR GLN ASN THR SER ASN MET ASN ALA GLY GLY LEU PHE GLY ALA LYS PRO GLN ASN THR THR ALA THR THR GLY GLY LEU PHE GLY SER LYS PRO GLN GLY SER THR THR ASN GLY GLY LEU PHE GLY SER GLY THR GLN ASN ASN ASN THR LEU GLY GLY GLY GLY LEU PHE GLY GLN SER GLN GLN PRO GLN THR ASN THR ALA PRO GLY LEU GLY ASN THR VAL SER THR GLN PRO SER PHE ALA TRP SER LYS PRO SER THR GLY SER

Supporting References

(1) Rubinstein M and Colby R 2003 Polymer physics *OUP Oxford*

(2) Sevick E 1996 *Macromolecules* 29:69526958

(3) Flory P 1971 Statistical mechanics of chain molecules. *Macmillan*
